# A Fatal Case of Rupture of the Bladder, from Urinary Calculi, in an Ox

**Published:** 1828-12

**Authors:** John Cook Bennett

**Affiliations:** Circleville


ART. II.—*A fatal case of rupture of the bladder, from Urinary Calculi, in an Ox.* Communicated in a letter from Dr. JOHN COOK BENNETT, of Circleville, to Dr. Jedediah Cobb, of Cincinnati.

*Circleville, Ohio, Dec. 2d*, 1828.

DEAR SIR:—

I send you some specimens of Urinary Calculi taken from the kidneys and bladder of an ox. The animal was a steer, two years old last spring, belonging to the Rev. Joseph Hays, of this county. From a calf, he at times appeared to be ill, and Mr. Hays supposed that he was afflicted with the colic, and gave him common salt, which appeared to afford temporary relief. To these attacks, as they were generally slight and of short duration, his owner paid but little personal attention. The last was upon the 15th November, ultimo.

In this, the animal expressed his sufferings by stamping with his hind feet, switching his tail, and making ineffectual efforts to void urine. There was a considerable loss of appetite for food and drink upon the first day of the attack, and afterwards it became total. With these symptoms he continued until the 17th. At this time he would often turn his head to his side and loins, as though there was pain in those regions. He lay the most of the time. Fever was quite apparent, and towards the last, very high. On the 18th, he expired in agony.

On opening the body, it was found that the bladder had been ruptured at its fundus. The opening was five inches in length. There were about ten gallons of urine in the cavity of the abdomen. Three small stones, about the size of a grain of wheat, were found in the neck of the bladder. The whole bladder and the muscles attached to its neck, to about half their length, were much inflamed. The left kidney had the appearance of having been in a very high state of inflammation, and its envelope adhered so closely, that it was with much difficulty removed. Its lobes and cavities were considerably enlarged, but at this advanced stage, it exhibited fewer marks of inflammation than of gangrene. The omentum and the fatty deposits about the kidneys, were somewhat inflamed. A portion of the fat had assumed the appearance of jelly. The right kidney was a little inflamed, and there were three or four small stones in one of its cavities. The stones contained in the left kidney, were mixed with sand and gravel, the whole weighing near an ounce and a half. The largest were of the size of hazle nuts. Those found in the bladder and right kidney, were perfectly clean and smooth. A part of the stones I send you for chemical examination. They are in the same condition in which they were taken from the ox.

Yours Respectfully,

John Cook Bennett.

Jedediah Cobb, M. D.

Editorial Note.—Dr. Cobb having favoured me with the specimens received from Dr. Bennett, I submitted them to examination, and will here state both the experiments and their results.

I. *Physical characters.* The calculi are of various sizes, from millet seeds up to hazle nuts; quite amorphous; some parts of the surface smooth, not (apparently) from friction; but, generally, rough with adhering particles; the outside of a dull russet colour—inside a pale yellow; friable under hard pressure with the fingers; more or less in concentric laminæ and cellular, like certain masses of tufa; on the whole, resembling irregular and loose masses of dry, yellowish clay; the smell offensive, and in some degree urinous. Under the blow pipe emitting the same odour, swelling up, blackening, burning with flame, sending forth a blue smoke, and disappearing before the continued application of heat.

II. *Chemical characters.* The specimens were subjected to experiments which afforded the following results:

1. They were insoluble or nearly insoluble in water, in which they swam, when thrown into it in lumps, until all their atmospheric air was expelled.

2. Thrown into a solution of potash, not *entirely* divested of carbonic acid, their dull yellow colour changed to a deeper and brighter hue, with a teint of green. During the process of dissolution, which continued till they were nearly dissolved, they sent out through the supernatent liquor, beautiful yellow clouds. When nitric acid was added to the solution, this colour was destroyed and a copious white precipitate instantly appeared.

3. When heated with nitric acid, a beautiful carmine red was generated.

4. When heated with caustic potash, they emitted the same smell as when under the blow pipe. The odour of ammonia was not perceptible; nor did white clouds (muriate of ammonia) show themselves, on blending the fumes of muriatic acid, from those arising from the mixture.

5. By a solution of carbonate of potash they were but slightly acted on; but in proportion as any dissolution took place the yellow colour was heightened.

From the foregoing experiments is appears, that these calculi are composed almost entirely of *lithic acid.* Their habitudes under the blow pipe, and when treated with a solution of potash, and with nitric acid, sufficiently demonstrate the presence of lithic acid; while their sparing solubility in water, and the results obtained by treating them with carbonate of potash, and fusing them with caustic potash, indicate the absence of ammonia.

Dr. Prout in his excellent work on urinary disorders, which should be in the hands of every physician, enumerates thirteen species of calculus, as known to the profession, and places the lithic acid at the head. This acid, indeed, constitutes alone a great portion of all the calculi which have been examined, while in its union with ammonia, or in its alternation with other ingredients, it enters more extensively into this class of morbid productions, than any other substance. Out of 823 specimens, the analysis of which is given by Dr. Prout, 98 were composed of pure lithic acid, while it entered as an element into 400 more. It seems to be in a great number of cases, the nucleus about which the phosphates and other materials arrange themselves, and but for which they would not be deposited. Thus, in the opinion of Dr. Prout, it is directly or indirectly the cause of at least two thirds of all the calculi that are formed, in the urinary organs of man. The lithic acid diathesis is the most frequent of all the calculous habits of body. It is in this diathesis, and this only perhaps, that the alkalies and alkaline earth are real, though not infallible antidotes.

To what extent our domestic animals are subject to urinary calculi, is far, I presume, from being known. Indeed, we are reprehensibly inattentive to all their diseases from the study of which we might, by means of sound analogy, derive many lessons of usefulness as physicians. Village and country practitioners especially, attached as many of them are, to rural occupations, and favoured with fine opportunities for observation, might by a diligent attention to the disorders of our domestic animals, and the careful dissection of those which die, contribute many useful facts to the departments of ætiology and pathology; and I strongly recommend Dr. Bennett's example to all the country physicians of the Western states.

